# Relationship between the presence of dedicated doctors in rapid response systems and patient outcome: a multicenter retrospective cohort study

**DOI:** 10.1186/s12931-021-01824-7

**Published:** 2021-08-26

**Authors:** Hyung-Jun Kim, Kyeongman Jeon, Byung Ju Kang, Jong-Joon Ahn, Sang-Bum Hong, Dong-Hyun Lee, Jae Young Moon, Jung Soo Kim, Jisoo Park, Jae Hwa Cho, Sang-Min Lee, Yeon Joo Lee

**Affiliations:** 1 Division of Pulmonary and Critical Care Medicine, Department of Internal Medicine, Seoul National University Bundang Hospital, Seongnam, Republic of Korea; 2 Department of Critical Care Medicine, Samsung Medical Center, Sungkyunkwan University School of Medicine, Seoul, Republic of Korea; 3 Department of Internal Medicine, Ulsan University Hospital, University of Ulsan College of Medicine, Ulsan, Republic of Korea; 4 Department of Pulmonary and Critical Care Medicine, Asan Medical Center, University of Ulsan College of Medicine, Seoul, Republic of Korea; 5 Division of Pulmonary and Critical Care Medicine, Department of Internal Medicine, Dong-A University College of Medicine, Busan, Republic of Korea; 6 Division of Pulmonary and Critical Care Medicine, Department of Internal Medicine, Chungnam National University Hospital, Chungnam National University College of Medicine, Daejeon, Republic of Korea; 7 Division of Pulmonary and Critical Care Medicine, Department of Internal Medicine, Inha University Hospital, Inha University School of Medicine, Incheon, Republic of Korea; 8 Division of Pulmonology, Department of Internal Medicine, CHA University, CHA Bundang Medical Center, Seongnam, Republic of Korea; 9 Division of Pulmonology, Department of Internal Medicine, Gangnam Severance Hospital, Yonsei University College of Medicine, Seoul, Republic of Korea; 10 Division of Pulmonary and Critical Care Medicine, Department of Internal Medicine, Seoul National University Hospital, Seoul National University College of Medicine, Seoul, Republic of Korea

**Keywords:** Hospital rapid response system, Propensity score, Physicians, Mortality

## Abstract

**Background:**

Rapid response systems (RRSs) improve patients’ safety, but the role of dedicated doctors within these systems remains controversial. We aimed to evaluate patient survival rates and differences in types of interventions performed depending on the presence of dedicated doctors in the RRS.

**Methods:**

Patients managed by the RRSs of 9 centers in South Korea from January 1, 2016, through December 31, 2017, were included retrospectively. We used propensity score-matched analysis to balance patients according to the presence of dedicated doctors in the RRS. The primary outcome was in-hospital survival. The secondary outcomes were the incidence of interventions performed. A sensitivity analysis was performed with the subgroup of patients diagnosed with sepsis or septic shock.

**Results:**

After propensity score matching, 2981 patients were included per group according to the presence of dedicated doctors in the RRS. The presence of the dedicated doctors was not associated with patients’ overall likelihood of survival (hazard ratio for death 1.05, 95% confidence interval [CI] 0.93‒1.20). Interventions, such as arterial line insertion (odds ratio [OR] 25.33, 95% CI 15.12‒42.44) and kidney replacement therapy (OR 10.77, 95% CI 6.10‒19.01), were more commonly performed for patients detected using RRS with dedicated doctors. The presence of dedicated doctors in the RRS was associated with better survival of patients with sepsis or septic shock (hazard ratio for death 0.62, 95% CI 0.39‒0.98) and lower intensive care unit admission rates (OR 0.53, 95% CI 0.37‒0.75).

**Conclusions:**

The presence of dedicated doctors within the RRS was not associated with better survival in the overall population but with better survival and lower intensive care unit admission rates for patients with sepsis or septic shock.

**Supplementary Information:**

The online version contains supplementary material available at 10.1186/s12931-021-01824-7.

## Background

Rapid response systems (RRS) are staffed by critical care experts and aimed to identify hospitalized patients at risk of rapid deterioration, enabling suitable interventions to be delivered before a catastrophic event [1]. Unlike the traditional code team, RRS are activated before a code situation occurs, as cardiac arrests are commonly preceded by premonitory signs and symptoms [2]. RRS have been widely deployed after the 100,000 Lives Campaign to reduce the number of preventable deaths [3]. Their implementation can reduce hospital mortality rates and non-intensive care unit (ICU) cardiopulmonary arrests [4].

The RRS teams usually include medical doctors, nurses, respiratory therapists, or pharmacists [5]. However, the optimal composition of these teams is controversial, and it may differ according to staff and resource availability at each center [6, 7]. Some RRS have dedicated doctors that do not have any other clinical obligations, while others include only dual appointment doctors whose primary duties are outside of the RRS [8]. Nurse practitioners are a reasonable substitute for medical doctors in some emergency care activities, suggesting hospitals may consider the cost-effectiveness of including dedicated doctors in the RRS, as some of their duties can be effectively performed by nurses [9].

Nevertheless, whether the presence of dedicated doctors in the RRS leads to better outcomes remains controversial. Actions such as those of lung care, oxygen supplementation, or fluid administration can be effectively performed by nurses without doctors on duty; thus, the presence of dedicated doctors within the RRS may not directly influence patient outcomes [10]. However, interventions such as central line insertion and endotracheal intubation can be more effectively performed when a physician has direct contact with the deteriorating patient. It would be considerably easier to recommend the optimal composition of the RRS if patient outcomes in the presence of dedicated doctors in the RRS are evaluated. In this study, we aimed to evaluate the differences in patient outcomes when dedicated doctors were present in the RRS. This is the first multicenter study to investigate this issue.

## Methods

### Data source and patient selection

The study participants were included in a nationwide multicenter retrospective cohort of nine RRS-operating referral centers in South Korea. Adult (aged ≥ 18 years) patients detected by any of these RRS from January 1, 2016, through December 31, 2017, were included. Each hospital had its own RRS activation criteria, including abnormal vital signs, mental status change, airway compromise, chest discomfort, extreme values of laboratory findings, or subjective concern expressed by the attending medical staff (details of these criteria per hospital are included in Additional file 1). Patients who activated the RRSs from the emergency or outpatient department, the ICU, or transferred to another hospital after RRS activation were excluded from this cohort.

The cohort data included information on patient demographic characteristics, comorbidities, location and time of the RRS activation, early warning scores such as the Modified Early Warning Score (MEWS) at the time of RSS activation [11], laboratory findings, types of interventions performed, and patient outcomes including mortality. The data were extracted retrospectively after reviewing the electronic medical records by trained nurses in each hospital. The development of this cohort was approved by the Institutional Review Board of each center, and the need for informed consent was waived due to the observational nature of the study and the use of anonymized data. Our study was conducted in accordance with the amended Declaration of Helsinki.

For propensity score-matched analysis, patients with missing data on the variables of interest, and those who remained hospitalized as of 31 December 2017, were excluded. We also excluded patients for whom the reason for RRS activation was unknown, as such patients are associated with high heterogeneity, which may affect propensity score matching.

### Definition of the propensity score and study outcomes

To control the impact of confounding factors on patients’ assignment to a hospital with dedicated doctors in the RRS, we used propensity score-matched analysis [12]. The propensity score was defined as the patients’ probability of being hospitalized at a center with dedicated doctors in the RRS. A “dedicated doctor” was defined as a medical doctor with expertise in critical care that worked exclusively for the RRS without any other clinical obligations. A dedicated doctor was present in three of nine centers.

The primary outcome was the likelihood of survival after RRS activation. The secondary outcome was the incidence of interventions performed after RRS activation, confined to those performed in the general ward before ICU admission. These interventions were grouped into 18 categories: advanced cardiovascular life support, extracorporeal membrane oxygenation, kidney replacement therapy, intubation, mechanical ventilation, bilevel positive airway pressure use, high-flow nasal cannula use, vasopressors use, bronchoscopy, arterial line insertion, central line insertion, portable sonography, computed tomography (CT), transfusion, antibiotics use, general management consultation, do not resuscitate (DNR) consultation, and ICU admission.

### Variable selection, balance assessment, and treatment effect estimates

Variables with a possible effect on both treatment assignment (detected by RRS with vs. without dedicated doctors) and the primary outcome (likelihood of survival) were included in the matching process [13]. This included age, sex, body mass index, comorbidities, MEWS, location of RRS activation, and reason for the RRS activation. For propensity score matching, 1:1 matching without replacement using the nearest neighbor method was used with the caliper of 0.2 [13, 14]. Standardized mean differences (SMDs) were calculated before and after matching to assess the balance of measured covariates [15].

Given the matched nature of this study, treatment effects were estimated using methods appropriate for paired samples [15, 16]. We used Cox regression for matched pairs to assess in-hospital survival outcomes, and hazard ratios (HRs) were calculated stratified [15]. For binary outcomes, we used conditional logistic regression analysis, in which odds ratios (ORs) were calculated [15].

### Sensitivity analysis

Due to the heterogeneity of RRS activation triggers, we included patients with sepsis or septic shock in sensitivity analyses, as this patient group is homogenous in its requirement of early intervention [17, 18]. Sepsis and septic shock were defined according to the Third International Consensus Definitions for Sepsis and Septic Shock [19]. As in our main analysis, patients were matched, the balance was assessed, and the treatment effect was estimated.

### Other statistical considerations

Categorical variables were presented as counts with percentages, and continuous variables were presented as means with standard deviations or 95% confidence intervals (CI). SMD of < 0.1 was considered negligible [15, 20]. Propensity score matching was performed with the “MachIt” package in R version 4.0.3 (The R Foundation for Statistical Computing, Vienna, Austria) [21].

## Results

### Patient characteristics before matching

**Fig. 1:**
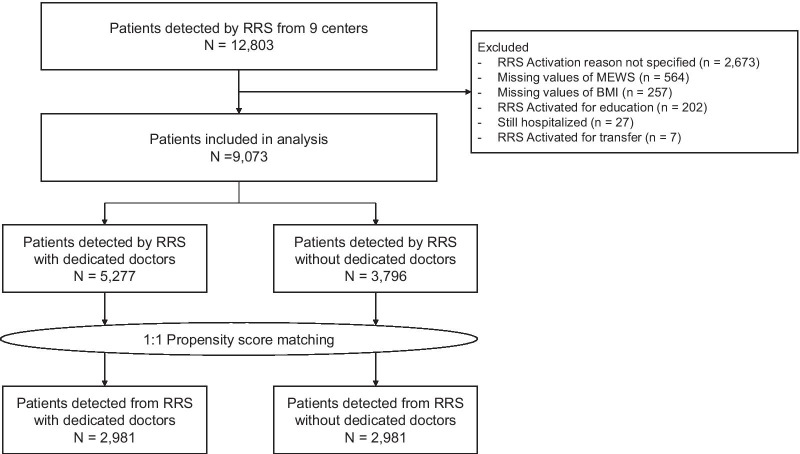
Flowchart of the patient selection process. Abbreviations: BMI, body mass index; MEWS, modified early warning score; RRS, rapid response system

During the study period, 12,803 patients were included in the retrospective multicenter cohort. Patients with missing data, those who remained hospitalized as of 31 December 2017, and those with an unknown reason for RRS activation were excluded. Finally, 9,073 patients were included in this study. Among them, 5277 patients (58.2%) were detected by the RRS with dedicated doctors (Fig. 1).

Patients detected by the RRS with dedicated doctors were younger (mean age 62.90 vs 67.90, SMD = 0.348) than those detected by the RSS without any dedicated doctors; however, the sex (female sex 39.2% vs 43.3%, SMD = 0.084) and body mass index (mean 22.38 vs 22.28, SMD = 0.021) compositions of both groups were similar. Patients detected by the RRS with dedicated doctors had a higher incidence of solid (46.9% vs 30.6%, SMD = 0.338) and hematologic (15.4% vs 4.9%, SMD = 0.353) malignancies, and that of organ transplant (5.0% vs 1.9%, SMD = 0.170), but lower incidence of diabetes mellitus (24.9% vs 29.6%, SMD = 0.106), chronic kidney disease (8.8% vs 12.7%, SMD = 0.126), and cerebrovascular disease (6.9% vs 16.8%, SMD = 0.309) than did those detected by the RRS without any dedicated doctors. Similarly, the former group was more likely to have higher MEWS scores (mean 4.75 vs 4.03, SMD = 0.326) and be detected at the medical department (78.3% vs 64.3%, SMD = 0.322) than was the latter group. The reasons for RRS activation differed significantly between the groups (SMD = 0.499) (Table 1).

**Table 1:** Characteristics of patients before and after propensity score matching

Variables	Before matching			After matching		
	RRS with dedicated doctor n = 5277	RRS without dedicated doctor n = 3796	SMD	RRS with dedicated doctor n = 2981	RRS without dedicated doctor n = 2981	SMD
Age, years	62.90 ± 14.36	67.90 ± 14.39	0.348	66.32 ± 13.46	66.83 ± 14.57	0.037
Female sex	2067 (39.2)	1643 (43.3)	0.084	1203 (40.4)	1241 (41.6)	0.026
Body mass index, kg/m^2^	22.38 ± 4.62	22.28 ± 4.43	0.021	22.26 ± 4.7	22.35 ± 4.48	0.020
Comorbidities						
Solid malignancy	2473 (46.9)	1163 (30.6)	0.338	1117 (37.5)	1069 (35.9)	
Cardiovascular disease	1329 (25.2)	892 (23.5)	0.039	777 (26.1)	733 (24.6)	0.033
Diabetes mellitus	1315 (24.9)	1125 (29.6)	0.106	821 (27.5)	830 (27.8)	0.034
Hematologic malignancy	811 (15.4)	185 (4.9)	0.353	197 (6.6)	182 (6.1)	0.007
Chronic lung disease	715 (13.5)	577 (15.2)	0.047	503 (16.9)	474 (15.9)	0.021
Chronic HBP disease	602 (11.4)	370 (9.7)	0.054	311 (10.4)	302 (10.1)	0.026
Chronic kidney disease	463 (8.8)	481 (12.7)	0.126	353 (11.8)	359 (12.0)	0.010
Cerebrovascular disease	364 (6.9)	636 (16.8)	0.309	320 (10.7)	384 (12.9)	0.006
Organ Transplantation	263 (5)	72 (1.9)	0.170	77 (2.6)	67 (2.2)	0.067
Gastrointestinal disease	173 (3.3)	181 (4.8)	0.076	125 (4.2)	129 (4.3)	0.022
Thyroid disease	135 (2.6)	123 (3.2)	0.041	87 (2.9)	84 (2.8)	0.007
MEWS	4.75 ± 2.16	4.03 ± 2.23	0.326	4.28 ± 2.06	4.21 ± 2.26	0.030
Department			0.322			0.034
Medical	4130 (78.3)	2439 (64.3)		2127 (71.4)	2082 (69.8)	
Surgical	1117 (21.2)	1345 (35.4)		841 (28.2)	887 (29.8)	
Obstetrics	30 (0.6)	12 (0.3)		13 (0.4)	12 (0.4)	
Location			0.014			0.014
General ward	5181 (98.2)	3726 (98.2)		2925 (98.1)	2923 (98.1)	
Examination unit	69 (1.3)	48 (1.3)		40 (1.3)	40 (1.3)	
Dialysis unit	13 (0.2)	12 (0.3)		6 (0.2)	8 (0.3)	
Others	14 (0.3)	10 (0.3)		10 (0.3)	10 (0.3)	
Reason for activation			0.499			0.098
Respiratory distress	2452 (46.5)	1797 (47.3)		1518 (50.9)	1462 (49)	
Hypovolemic shock	483 (9.2)	164 (4.3)		156 (5.2)	149 (5)	
Arrhythmias	478 (9.1)	253 (6.7)		241 (8.1)	238 (8)	
Septic shock	402 (7.6)	203 (5.3)		181 (6.1)	182 (6.1)	
Altered mental status	381 (7.2)	144 (3.8)		140 (4.7)	128 (4.3)	
Sepsis	292 (5.5)	115 (3)		118 (4)	110 (3.7)	
High blood pressure	286 (5.4)	673 (17.7)		276 (9.3)	361 (12.1)	
Metabolic acidosis	271 (5.1)	230 (6.1)		183 (6.1)	185 (6.2)	
Cardiac arrest	131 (2.5)	153 (4)		117 (3.9)	117 (3.9)	
Cardiogenic shock	47 (0.9)	28 (0.7)		23 (0.8)	26 (0.9)	
Anaphylactic shock	40 (0.8)	10 (0.3)		14 (0.5)	10 (0.3)	
Obstructive shock (PTE)	14 (0.3)	26 (0.7)		14 (0.5)	13 (0.4)	

Numbers are presented as count (percentage) or mean ± standard deviation

HBP, hepato-biliary-pancreatic; MEWS, modified early warning score; PTE, pulmonary thromboembolism; RRS, rapid response system; SMD, standardized mean difference

### Propensity score-matched analysis

All variables presented in Table 1 were likely to affect both treatment assignment (detection by the RRS with dedicated doctors) and outcome (likelihood of survival) and were thus used to obtain the propensity score. After 1:1 matching, 2981 patients were included per group (RSS with vs. without dedicated doctors) (Fig. 1).

After matching, the groups were well-balanced with a SMD of < 0.1 for all variables used to obtain the propensity score (Table 1). For details in changes to the SMDs per variable, see Additional file 2: Figure S1. The distribution of the propensity scores was similar between the groups after matching (Additional file 2: Figure S2).

### Impact on patient survival

**Fig. 2:**
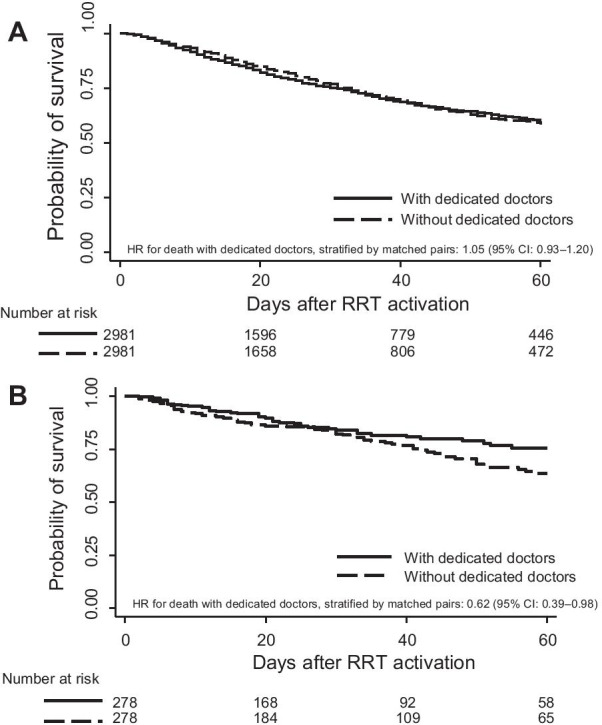
Probability of survival according to the presence of dedicated doctors in the rapid response system. **A** Main analysis regardless of the reason for rapid response system activation. **B** Sensitivity analysis with patients diagnosed as sepsis or septic shock

After propensity score matching, there was no between-group difference in overall survival outcomes (HR of death 1.05, 95% CI 0.93–1.2) (patients in the RSS with vs. without dedicated doctors). The Kaplan–Meier curve also showed a similar probability of survival for both groups after RSS activation (Fig. 2A).

### Impact on types of interventions

**Fig. 3:**
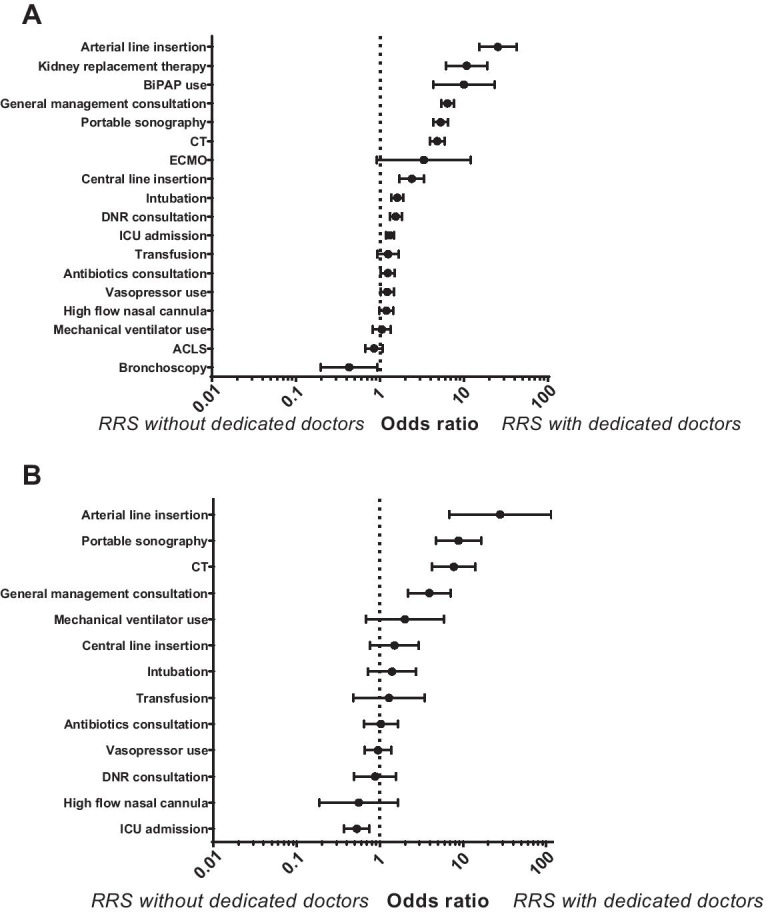
Interventions performed according to the presence of dedicated doctors in the rapid response system. **A** Main analysis regardless of the reason for rapid response system activation. **B** Sensitivity analysis with patients diagnosed as sepsis or septic shock. Dots represent odds ratios and bars represent 95% confidence intervals. Abbreviations: ACLS, advanced cardiovascular life support; BiPAP, bilevel positive airway pressure; CT, computed tomography; DNR, do not resuscitate; ECMO, extracorporeal membrane oxygenation; ICU, intensive care unit

A total of 10,488 interventions were performed in the general ward before ICU admission. General management consultation was the most common intervention performed (45.4%), followed by portable sonography (7.2%), CT imaging evaluation (6.7%), and intubation (6.3%) (Additional file 2: Table S1). Patients detected by the RRS with dedicated doctors tended to undergo more interventions before ICU admission than those detected by the RRS without any dedicated doctors. The interventions were arterial line insertion (OR 25.33, 95% CI 15.12–42.44), kidney replacement therapy (OR 10.77, 95% CI 6.10–19.01), bilevel positive airway pressure use (OR 10.00, 95% CI 4.32–23.15), general management consultation (OR 6.37, 95% CI 5.33–7.60), portable sonography (5.26, 95% CI 4.30–6.45), CT imaging evaluation (OR 4.81, 95% CI 3.93–5.90), central line insertion (OR 2.40, 95% CI 1.71–3.36), intubation (OR 1.60, 95% CI 1.36–1.90), DNR consultation (OR 1.54, 95% CI 1.30–1.82), and ICU admission (OR 1.31, 95% CI 1.18–1.46). Conversely, these patients underwent less bronchoscopy (OR 0.43, 95% CI 0.20–0.94) (Fig. 3A).

### Sensitivity analysis

A total of 1012 patients with sepsis or septic shock were selected for sensitivity analysis, including 694 patients (68.6%) detected by the RRS with dedicated doctors. After 1:1 matching using the covariates, 278 patients were included per group, with well-balanced characteristics in both groups (Table 2). The quick Sequential Organ Failure Assessment score showed a median of 1 point (interquartile range 1–2), with no significant difference between the two groups (*P* = 0.189 by Wilcoxon signed-rank test). The score revealed poor performance to predict the probability of in-hospital death (Harrell’s c-index 0.58, 95% CI 0.52–0.63). Cox regression analysis by matched pairs revealed that patients detected by the RRS with dedicated doctors revealed better survival rates than those detected by the RSS without any dedicated doctors (HR for death 0.62 with 95% CI 0.30–0.98) (Fig. 2B).

**Table 2:** Characteristics of patients diagnosed as sepsis or septic shock before and after propensity score matching

Variables	Before matching			After matching		
	RRS with dedicated doctor n = 694	RRS without dedicated doctor n = 318	SMD	RRS with dedicated doctor n = 278	RRS without dedicated doctor n = 278	SMD
Age, years	62.25 ± 13.2	67.08 ± 13.71	0.359	65.76 ± 12.25	66.18 ± 13.71	0.032
Female sex	287 (41.4)	150 (47.2)	0.117	131 (47.1)	128 (46)	0.022
BMI, kg/m^2^	22.35 ± 4.24	22.51 ± 3.81	0.040	22.57 ± 4.36	22.63 ± 3.82	0.015
Comorbidities						
Solid malignancy	347 (50)	107 (33.6)	0.336	101 (36.3)	107 (38.5)	0.045
Diabetes mellitus	175 (25.2)	99 (31.1)	0.132	79 (28.4)	85 (30.6)	0.047
Hematologic malignancy	154 (22.2)	24 (7.5)	0.421	31 (11.2)	23 (8.3)	0.097
Cardiovascular disease	134 (19.3)	54 (17)	0.060	49 (17.6)	49 (17.6)	< 0.001
Chronic HBP disease	81 (11.7)	47 (14.8)	0.092	36 (12.9)	45 (16.2)	0.092
Chronic kidney disease	45 (6.5)	37 (11.6)	0.180	30 (10.8)	31 (11.2)	0.012
Chronic lung disease	40 (5.8)	30 (9.4)	0.139	21 (7.6)	21 (7.6)	< 0.001
Cerebrovascular disease	40 (5.8)	49 (15.4)	0.317	26 (9.4)	31 (11.2)	0.059
Organ Transplantation	33 (4.8)	10 (3.1)	0.083	10 (3.6)	9 (3.2)	0.020
Gastrointestinal disease	18 (2.6)	29 (9.1)	0.281	13 (4.7)	18 (6.5)	0.078
Thyroid disease	15 (2.2)	13 (4.1)	0.111	8 (2.9)	10 (3.6)	0.041
MEWS	5.47 ± 2.3	5.05 ± 2.27	0.185	4.94 ± 2.14	5.09 ± 2.28	0.072
Department			0.295			0.049
Medical	566 (81.6)	224 (70.4)		202 (72.7)	208 (74.8)	
Surgical	124 (17.9)	94 (29.6)		76 (27.3)	70 (25.2)	
Obstetrics	4 (0.6)	0 (0)		–	–	
Location			0.054			< 0.001
General ward	693 (99.9)	318 (100)		278 (100)	278 (100)	
Others	1 (0.1)	0 (0)		–	–	

Numbers are presented as count (percentage) or mean ± standard deviation

HBP, hepato-biliary-pancreatic; MEWS, modified early warning score; RRS, rapid response system; SMD, standardized mean difference

For most interventions, no difference in incidence was detected between the groups due to a small number of affected patients. However, patients detected by the RRS with dedicated doctors tended to undergo more arterial line insertion (OR 28.00, 95% CI 6.83–114.70), portable sonography (OR 8.82, 95% CI 4.73–16.45), CT imaging evaluation (OR 7.75, 95% CI 4.25–14.14), and general management consultation (3.93, 95% CI 2.19–7.06), but less ICU admission (OR 0.53, 95% CI 0.37–0.75) than did those detected by the RRS without any dedicated doctors (Fig. 3B).

## Discussion

In this propensity score-matched multicenter retrospective cohort study, the overall likelihood of survival among patients detected by the RRS was similar regardless of the presence of dedicated doctors. However, the presence of dedicated doctors in the RRS was associated with more frequent interventions such as arterial line insertion and kidney replacement therapy. Furthermore, patients with sepsis or septic shock in the RSS with dedicated doctors revealed a greater likelihood of survival and lower ICU admission rate than those in the RSS without any dedicated doctors.

Our study findings reflect previous controversies regarding the role of dedicated doctors in the RRS [8, 10, 22–24]. Previously reported incidence of cardiac arrest and ICU transfer was similar when RRS was led by either senior residents or ICU physicians [22]; similarly, the rate of in-hospital death and length of stay estimates were similar when the RRS was driven by intensivists, nurses, or house staff [23]. Furthermore, a previous systematic review of 29 studies suggested that the presence of a physician in the RRS did not affect the rates of in-hospital mortality or cardiopulmonary arrest [10].

Despite the increased incidence of interventions in the RRS in our study, similar survival outcomes can be explained by two different perspectives. First, in most circumstances, nurse-driven actions such as low-flow oxygen supplementation/adjustment may be sufficient in early-stage resuscitation. In our matched population, the most common reason for RRS activation was respiratory distress, affecting 2980 (50.0%) patients; however, only 599 (20.1%) of them required intubation. The predominance of low-flow oxygen titration after RRS activation has been previously reported [25]. Moreover, studies have shown that standard oxygen therapy may be non-inferior to high-flow oxygen supply [26] and that non-invasive ventilation may suffice, replacing invasive ventilation in acute respiratory failure [27]. Likewise, nurse-driven activities such as intravenous fluid or as-needed drug administration may suffice as primary management. In hypovolemic shock, which accounted for 305 (5.1%) patients in our matched population, intravenous fluid therapy is the mainstay of treatment [28]. The administration of intravenous antihypertensive medication is the primary standard-of-care treatment for high blood pressure [29], which was the second most common reason for RRS activation in our study (637 [10.7%] patients).

Second, interventions may be futile in the end stages of chronic illness. In our study, matched patients had underlying solid malignancy or hematologic malignancy in over 40% of cases. Although the proportion of end-stage disease could not be ascertained, metabolic acidosis and altered mental state due to end-stage processes are difficult to reverse despite interventions. Moreover, the increased frequency of a DNR consultation after RRS activation may have decreased the survival rate upon discharge [30].

This is the first study to report better survival outcomes of patients with sepsis or septic shock among those detected by the RRS with dedicated doctors than those detected by the RSS without such doctors. The recent Surviving Sepsis Campaign Bundle has introduced a 1-h bundle, which includes obtaining blood cultures, administrating antibiotics, and aggressively resuscitating the patient with intravenous fluid and vasopressors within 1 h of detection [17]. Moreover, invasive arterial pressure monitoring has been recommended to identify the specific anatomic origin of infection and decide whether emergent source control is required [18]. These recommendations are concordant with our study findings. Dedicated doctors underwent arterial line insertion for invasive blood pressure monitoring, checked sonography/CT for anatomic evaluation of infection, and provided general management consultation for the overall process of the 1-h bundle. These interventions may be beneficial to patients with sepsis or septic shock and may improve survival outcomes and lower the rates of ICU admissions [31].

Overall, the present findings suggest that the presence of dedicated doctors within the RSS may be helpful in particular situations, especially for those where the implementation of early bundle-based approaches is required. Considering the high incidence of sepsis among patients detected by the RRS [32], aggressive evaluation and intervention by a dedicated doctor should be recommended when either sepsis or septic shock is suspected. Future prospective studies are required to confirm the beneficial impact of dedicated doctors in the RRS on patients in need of bundle-based approaches.

This study had some limitations. First, some aspects of RRS in each center were not evaluated, such as staff communication skills or leadership during the RRS-activating event or the clarity of team members’ roles [8], all of which are potentially relevant. Second, this was a multicenter retrospective observational study based on South Korean patients. Although the propensity score matching method was used, it should be interpreted with caution. Further studies are required to generalize the results to other ethnic populations.

## Conclusions

The presence of dedicated doctors in the RRS was not associated with the overall patient survival estimates. However, compared with that without, the RRS with dedicated doctors was associated with more frequent interventions and was associated with improved survival outcomes and lesser ICU admission rates among patients with sepsis or septic shock.

## Supplementary Information

**Additional file 1:** Activation criteria of the rapid response systems according to each center (anonymized).

**Additional file 2: Figure S1.** Change of the values of standardized mean differences before and after propensity score matching. **Figure S2.** Distribution of the propensity scores before and after matching. **Table S1.** Overall incidence of interventions performed after the activation of the rapid response system in the matched population.

## Data Availability

The datasets used and/or analyzed during the current study are available from the corresponding author on reasonable request.

## Abbreviations

CI: Confidence interval
CT: Computed tomography
DNR: Do not resuscitate
HR: Hazard ratio
ICU: Intensive care unit
MEWS: Modified Early Warning Score
OR: Odds ratio
RRS: Rapid response system
SMD: Standardized mean difference
