# Supplementary material for: Relationship between the presence of dedicated doctors in rapid response systems and patient outcome: a multicenter retrospective cohort study
Source: Respir Res. 2021 Aug 26;22:236. doi: 10.1186/s12931-021-01824-7 (PMC8394678; doi:10.1186/s12931-021-01824-7)
Supplement: Supplementary file 1 — Additional file 1: Activation criteria of the rapid response systems according to each center (anonymized). [file 12931_2021_1824_MOESM1_ESM.pdf]

Activation criteria of the rapid response systems according to each center (anonymized)

Center A

A. Calling criteria

- Respiratory: respiratory rate ≥30, SpO_2 <85% for 5 minutes, pH <7.3 & $PaCO_2$ >50, stridor, use of accessary respiratory muscles
- Cardiovascular: systolic blood pressure <85, heart rate >130, acute chest pain, symptomatic arrhythmia
- Neurologic: altered mental status of sudden onset, unexplained agitation, seizure
- Others: bedside nurse concern about overall deterioration

B. Screening criteria

- MEWS (modified early warning score) ≥7

Center B

A. Calling criteria

- Airway: threatened, stridor
- Breathing: respiratory rate <6 or >30, SpO_2 <90% on Venturi 40% or O_2 6 L/min
- Circulation: heart rate <40 or >140, systolic blood pressure <90
- Neurology: sudden mental change, seizure
- Others: bedside nurse's concern about overall deterioration

B. Screening criteria

- Systolic blood pressure <86
- Sudden mental change or unexplained agitation
- Applying O_2 >9 L/min, or FiO_2 >35%
- Respiratory rate >27 or <8
- Unexplained heart rate >140 or <40
- Unexplained severe metabolic acidosis: pH <7.3, lactate >2 mmol/L, tCO_2 <12 mmol/L
- $PaCO_2$ >50 or PaO_2 ≤55
- Bedside nurse concern about overall deterioration

Center C

- respiratory rate >28 or <8

- heart rate >140 or <40

- systolic blood pressure <90

- ABGA: pH <7.3 or >7.6, HCO_3 <14 mmol/L, $PaCO_2$ >50, PaO_2 <55

- Lactate >30 mg/dL, tCO_2 <15 mmol/L, potassium >6 or <3 mmol/L

- SpO_2 <90%, O_2 >8 L/min

Center D

- Systolic blood pressure: <90

- Heart rate: <50, >140

- Respiratory rate: <10, >30

- Body temperature: <36°C, >39°C

- SpO_2: <90%

- ABGA: pH <7.25, $PaCO_2$ >50, PaO_2 <55

- Lactate >4 mmol/L

- tCO_2 <15 mmol/L

Center E

A. Calling criteria

- Airway: threatened airway, stridor, wheezing sound

- Breathing: respiratory rate ≤6 or ≥30, SpO_2 <90% on O_2 6 L/min or venturi mask 40%

- Circulation: heart rate <40 or ≥140, systolic blood pressure <90

- Neurology: sudden mental change, seizure

- Others: bedside nurse's concern about overall deterioration

B. Screening criteria

- Airway: threatened airway, stridor, wheezing sound

- Breathing: respiratory rate ≤6 or ≥30, SpO_2 <90%, oxygen demand (over nasal prong 6 L/min or venturi mask 40%)

- Circulation: heart rate <40 or ≥140, systolic blood pressure ≤85

- Neurology: sudden mental change, seizure

- Others: pH ≤7.33, HCO_3 ≤15 mmol/L, lactate ≥2 mmol/L, potassium ≥6 mmol/L, tCO_2 ≤15 mmol/L, glucose ≤50 mg/dL, ICU discharge patients

Center F

A. Calling criteria

- Respiratory: respiratory rate ≤8 or ≥28, SpO_2 ≤90% for 5 minutes, dyspnea of sudden onset
- Cardiovascular: heart rate ≤40 or ≥130, systolic blood pressure ≤80 or ≥200, systolic blood pressure 80–90 with symptom, chest pain not responsive to sublingual nitroglycerin
- Neurologic: altered consciousness of sudden onset, sudden paralysis of face or extremities, new onset seizure, prolonged agitation (≥10 minutes) not fully explained by medical conditions
- Others: color change of peripheral extremities, subjective judgement of attending physician or nurse

B. Screening criteria

- Heart rate: <41, >129
- Respiratory rate: <9, >27
- Systolic blood pressure: <81, >199
- SpO_2: <90%

Center G

A. Calling criteria

- Airway: airway obstruction sign, stridor
- Breathing: respiratory rate <8 or >30, pH <7.3 & $PaCO_2$ >60, SpO_2 <90% on facial mask or high flow nasal cannula
- Circulation: heart rate <40 or >140, systolic blood pressure <90, lactate >2 mmol/L
- Neurology: sudden mental change or unexplained agitation, seizure
- Nurse's concern about overall deterioration

B. Screening criteria

- based on NEWS (national early warning score)

Center H

A. Calling criteria

- Breathing: respiratory rate ≤8 or ≥30, SpO_2 <90% for 5 minutes on O_2 >5 L/min, $PaCO_2$ >50

- Circulation: systolic blood pressure <90, heart rate <40 or >130, acute chest pain

- Neurology: sudden mental change, seizure

- Others: bedside nurse's concern about overall deterioration

B. Screening criteria

- Breathing: respiratory rate ≤8 or ≥25, SpO_2 ≤90%, PaO_2 ≤55, $PaCO_2$ ≥50, pH ≤7.3, lactate ≥2 mmol/L, tCO_2 ≤12 mmol/L

- Circulation: systolic blood pressure ≤90, heart rate ≤40 or ≥130, acute chest pain

- Neurology: sudden mental change, seizure

- 48 hours after discharge from ICU

- High risk surgical patients

Center I

- mean blood pressure <60 or systolic blood pressure <90

- applying O_2 >9 L/min or venturi mask >35%

- respiratory rate >25 or <8

- heart rate >140 or <40

- glucose ≤50 mg/dL

- unexplained severe metabolic acidosis: pH<7.3 or lactate >2 mmol/L or tCO_2 <16 mmol/L

- PaO_2 ≤55 or $PaCO_2$ >50

- sudden mental change or unexplained agitation, unexplained seizures

- chest pain, upper airway obstruction sign like stridor

- bedside nurse concern about overall deterioration
