# Supplementary material for: Relationship between the presence of dedicated doctors in rapid response systems and patient outcome: a multicenter retrospective cohort study
Source: Respir Res. 2021 Aug 26;22:236. doi: 10.1186/s12931-021-01824-7 (PMC8394678; doi:10.1186/s12931-021-01824-7)
Supplement: Supplementary file 2 — Additional file 2: Figure S1. Change of the values of standardized mean differences before and after propensity score matching. Figure S2. Distribution of the propensity scores before and after matching. Table S1. Overall incidence of interventions performed after the activation of the rapid response system in the matched population. [file 12931_2021_1824_MOESM2_ESM.pdf]

Figure S1. Change of the values of standardized mean differences before and after matching

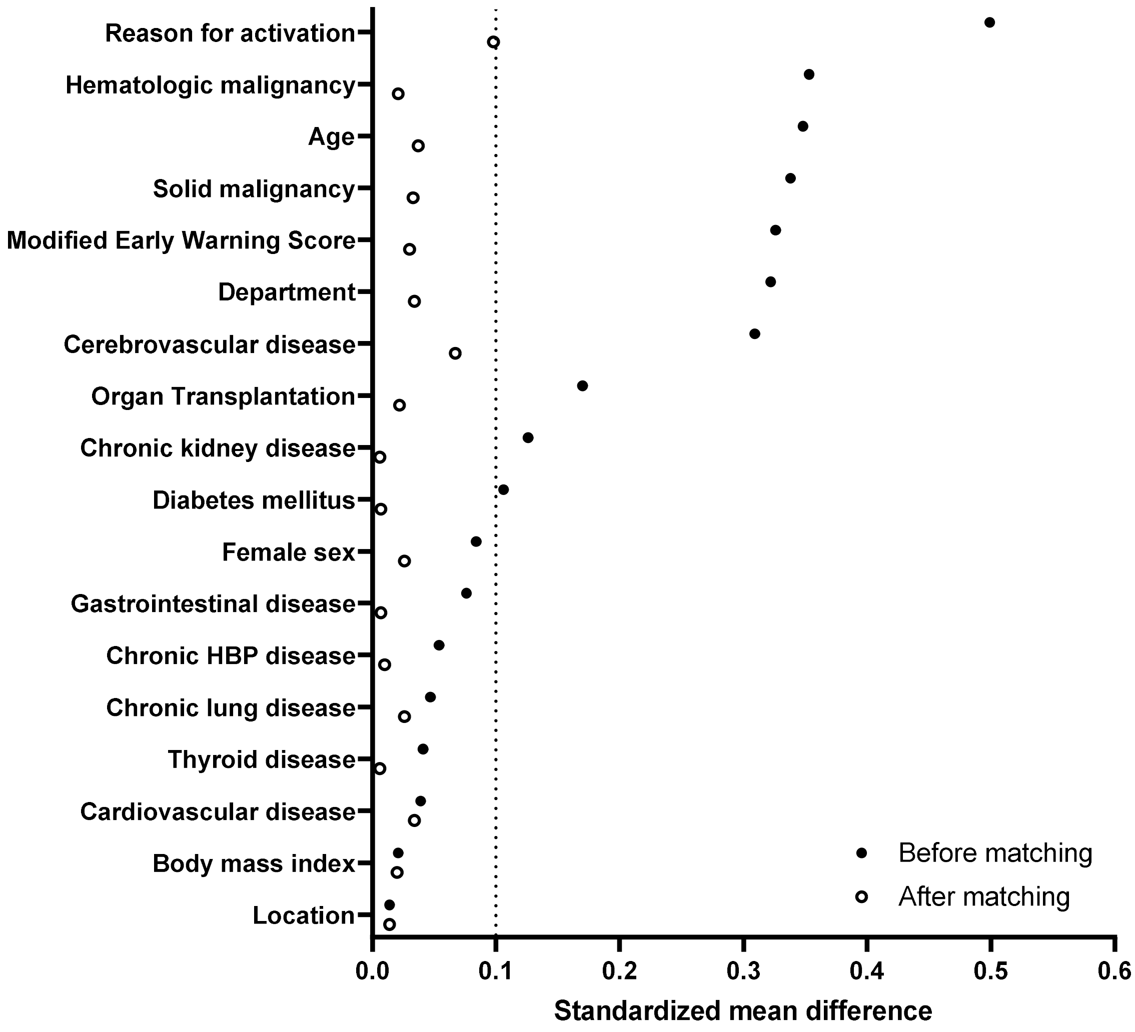

Abbreviation: HBP, hepato-biliary-pancreatic.

Figure S2. Distribution of the propensity scores before and after matching

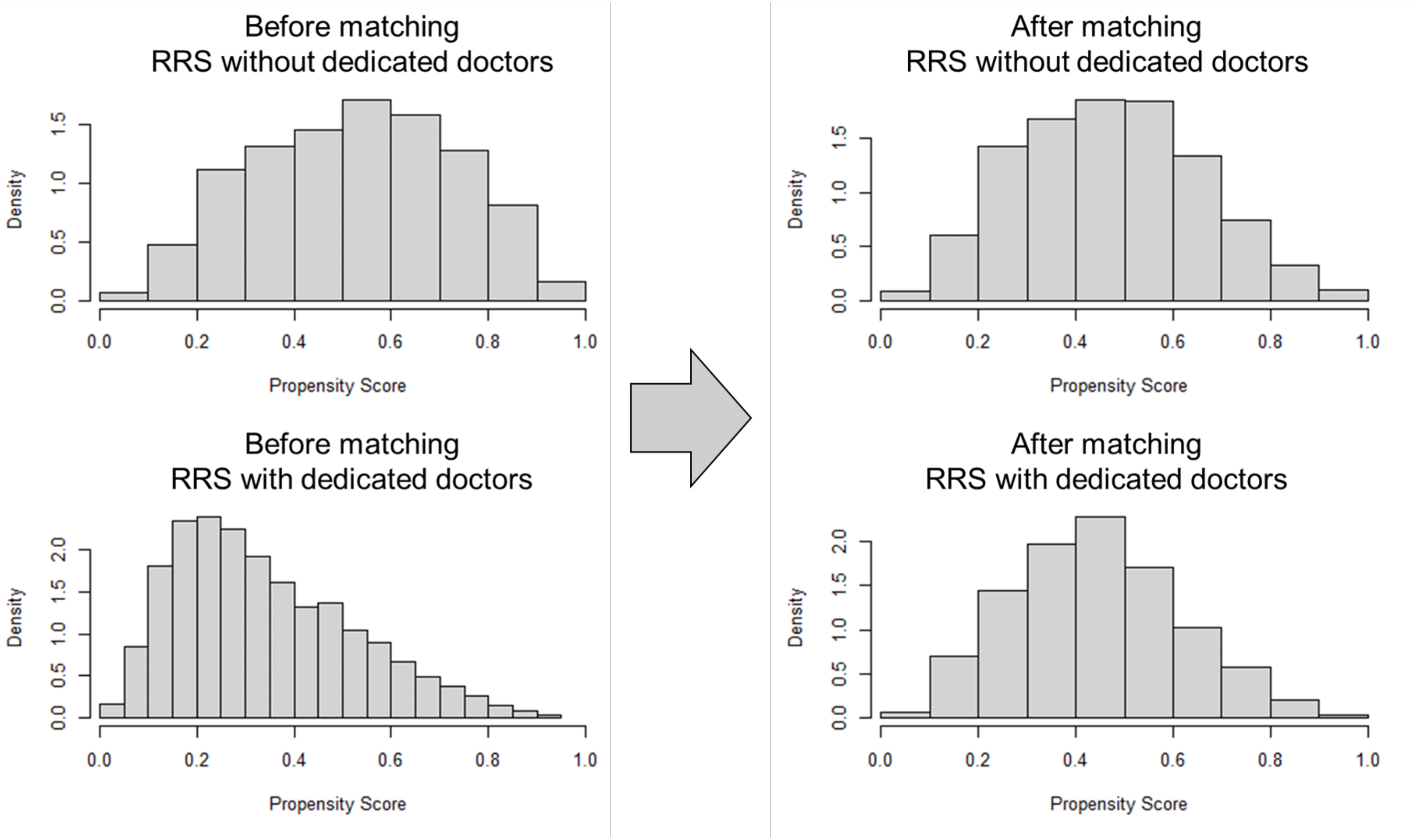

Abbreviations: RRS, rapid response system

Table S1. Overall incidence of interventions performed after the activation of the rapid response system in the matched population

Types of intervention	Number of interventions performed N=10,488
Treatment plan consultation	4766 (45.4)
Portable sonography	751 (7.2)
CT	699 (6.7)
Intubation	662 (6.3)
DNR consult	645 (6.1)
Vasopressor use	494 (4.7)
High flow nasal cannula	455 (4.3)
Antibiotics consultation	444 (4.2)
Arterial line insertion	403 (3.8)
ACLS	288 (2.7)
Mechanical ventilator use	250 (2.4)
Transfusion	192 (1.8)
Central line insertion	171 (1.6)
Kidney replacement therapy	157 (1.5)
BiPAP use	68 (0.6)
Bronchoscopy	30 (0.3)
ECMO	13 (0.1)

Numbers are presented as count (percentage).

Abbreviations: ACLS, advanced cardiovascular life support; BiPAP, bilevel positive airway pressure; CT, computed tomography; DNR, do-not-resuscitate; ECMO, extracorporeal membrane oxygenation.
